# Supplementary material for: Subgroup evaluation to understand performance gaps in deep learning-based classification of regions of interest on mammography
Source: PLOS Digit Health. 2025 Apr 8;4(4):e0000811. doi: 10.1371/journal.pdig.0000811 (PMC11978028; doi:10.1371/journal.pdig.0000811)
Supplement: S1 Table — (DOCX) [file pdig.0000811.s001.docx]

| **S1 Table: Comparison of performance of multiple standard convolutional neural network (CNN) models for binary patch classification on mammography.** | | | | | |
| --- | --- | --- | --- | --- | --- |
| **Model** | **Accuracy** | **AUC** | **Recall** | **Precision** | **F1 Score** |
| **VGG16** | 0.794 | 0.881 | 0.794 | 0.766 | 0.780 |
| **InceptionV3** | 0.906 | 0.967 | 0.886 | 0.907 | 0.897 |
| **ResNet50V2** | 0.918 | 0.968 | 0.912 | 0.909 | 0.910 |
| **ResNet152V2** | 0.926 | 0.975 | 0.927 | 0.912 | 0.919 |
| **Note:** AUC = Area Under the receiver operating characteristics Curve | | | | | |
