# Supplementary material for: Subgroup evaluation to understand performance gaps in deep learning-based classification of regions of interest on mammography
Source: PLOS Digit Health. 2025 Apr 8;4(4):e0000811. doi: 10.1371/journal.pdig.0000811 (PMC11978028; doi:10.1371/journal.pdig.0000811)
Supplement: S2 Table — (DOCX) [file pdig.0000811.s002.docx]

| **S2 Table: Classification Performance in Subgroups Stratified by Tissue Density on the Test Set** | | | | | | |
| --- | --- | --- | --- | --- | --- | --- |
| **Subgroups** | **Metrics** | **Density A** | **Density B** | **Density C** | **Density D** | **Overall** |
| **Overall** | AUC:  Recall:  Precision: | 0.977±0.013  0.924±0.040  0.825±0.069 | 0.982±0.004  0.942±0.012  0.936±0.014 | 0.966±0.005  0.920±0.012  0.906±0.013 | 0.953±0.021  0.899±0.046  0.882±0.047 | **0.975±0.003**  **0.927±0.008**  **0.912±0.010** |
| **Race** | | | | | | |
| White | AUC:  Recall:  Precision: | 0.984±0.011  0.920±0.048  0.787±0.068 | 0.978±0.008  0.936±0.023  0.933±0.020 | 0.962±0.011  0.907±0.026  0.895±0.024 | 0.955±0.006  0.882±0.013  0.870±0.014 | 0.972±0.005  0.918±0.013  0.902±0.016 |
| Black | AUC:  Recall:  Precision: | 0.973±0.010  0.924±0.031  0.846±0.039 | 0.983±0.005  0.941±0.019  0.938±0.019 | 0.967±0.011  0.924±0.020  0.907±0.022 | 0.949±0.008  0.900±0.016  0.874±0.019 | 0.976±0.005  0.931±0.012  0.914±0.015 |
| Other | AUC:  Recall:  Precision: | 0.988±0.015  0.953±0.077  0.838±0.142 | 0.988±0.007  0.953±0.026  0.940±0.030 | 0.972±0.011  0.930±0.025  0.924±0.023 | 0.949±0.009  0.923±0.017  0.910±0.016 | 0.978±0.007  0.938±0.020  0.926±0.020 |
| **Age Group** | | | | | | |
| <50 | AUC:  Recall:  Precision: | 0.984±0.011  0.921±0.066  0.847±0.086 | 0.977±0.011  0.939±0.027  0.940±0.027 | 0.966±0.010  0.919±0.021  0.915±0.019 | 0.951±0.006  0.869±0.013  0.909±0.013 | 0.970±0.007  0.922±0.014  0.919±0.016 |
| 50-60 | AUC:  Recall:  Precision: | 0.971±0.024  0.906±0.065  0.780±0.072 | 0.986±0.006  0.944±0.023  0.952±0.017 | 0.968±0.010  0.918±0.023  0.905±0.021 | 0.972±0.005  0.974±0.010  0.853±0.019 | 0.977±0.005  0.930±0.017  0.915±0.018 |
| 60-70 | AUC:  Recall:  Precision: | 0.985±0.007  0.936±0.036  0.880±0.041 | 0.980±0.007  0.934±0.022  0.931±0.023 | 0.965±0.016  0.927±0.035  0.906±0.035 | 0.937±0.015  0.859±0.027  0.911±0.029 | 0.976±0.006  0.932±0.017  0.916±0.022 |
| >70 | AUC:  Recall:  Precision: | 0.966±0.022  0.926±0.054  0.780±0.073 | 0.983±0.008  0.949±0.028  0.916±0.036 | 0.962±0.017  0.901±0.056 0.875±0.050 | 0.943±0.018  0.921±0.035  0.766±0.043 | 0.975±0.008  0.928±0.023  0.882±0.031 |
| **Pathology** | | | | | | |
| Cancer | AUC:  Recall:  Precision: | 1.000^†^  1.000^†^  1.000^†^ | 0.975±0.050  0.947±0.077  0.949±0.068 | 0.981±0.032  0.911±0.126  0.957±0.097 | 1.000^†^  1.000^†^  1.000^†^ | 0.980±0.023  0.938±0.063  0.957±0.052 |
| Benign | AUC:  Recall:  Precision: | 0.999±0.004  1.000^†^  0.972±0.045 | 0.981±0.015  0.949±0.039  0.955±0.030 | 0.969±0.024  0.949±0.042  0.926±0.049 | 0.994±0.004  0.966±0.024  0.965±0.023 | 0.977±0.010  0.955±0.022  0.943±0.027 |
| Never Biopsied | AUC:  Recall:  Precision: | 0.975±0.008  0.913±0.025  0.809±0.035 | 0.982±0.004  0.940±0.014  0.934±0.013 | 0.966±0.006  0.916±0.013  0.904±0.014 | 0.948±0.005  0.888±0.009  0.870±0.010 | 0.974±0.003  0.925±0.008  0.908±0.010 |
| **Image Findings** | | | | | | |
| Mass | AUC:  Recall:  Precision: | 0.996±0.003  0.979±0.033  0.820±0.073 | 0.984±0.013  0.957±0.031  0.932±0.036 | 0.973±0.013  0.934±0.033  0.889±0.044 | 0.936±0.014  0.952±0.016  0.852±0.028 | 0.980±0.008  0.949±0.022  0.896±0.029 |
| Calcification | AUC:  Recall:  Precision: | 0.987±0.010  0.965±0.041  0.829±0.076 | 0.979±0.010  0.942±0.026  0.926±0.034 | 0.969±0.013  0.933±0.027  0.903±0.033 | 0.967±0.006  0.954±0.012  0.875±0.016 | 0.974±0.008  0.939±0.018  0.904±0.022 |
| AD | AUC:  Recall:  Precision: | 0.873±0.222  0.595±0.343  1.000^†^ | 0.947±0.033  0.804±0.072  0.985±0.026 | 0.902±0.056  0.831±0.066  0.918±0.057 | 0.895±0.031  0.718±0.038  0.897±0.035 | 0.914±0.034  0.810±0.040  0.939±0.027 |
| Asymmetry | AUC:  Recall:  Precision: | 0.983±0.009  0.911±0.031  0.982±0.016 | 0.988±0.004  0.955±0.014  0.963±0.013 | 0.966±0.009  0.924±0.016  0.919±0.018 | 0.973±0.006  0.929±0.013  0.936±0.012 | 0.977±0.005  0.937±0.010  0.942±0.011 |
| **Total Count** |  | 1,666 (12.4%) | 4,921 (36.8%) | 6,226 (46.5%) | 577 (4.3%) | 13,390 (100%) |
| **Note:** The overall and subgroup AUC, recall, and precision averaged over 200 bootstrapped samples ± 95% confidence interval, randomly sized ranging in [500,1666] for patches with BI-RADS density A, [500,4921] for patches with BI-RADS density B, [500,6226] for patches with BI-RADS density C, and [500,577] for patches with BI-RADS density D.  AD = Architectural Distortion, AUC = Area Under the receiver operating characteristics Curve  ^†^: Cases within subgroup were all correctly classified, no confidence intervals applicable | | | | | | |
