# Supplementary material for: Subgroup evaluation to understand performance gaps in deep learning-based classification of regions of interest on mammography
Source: PLOS Digit Health. 2025 Apr 8;4(4):e0000811. doi: 10.1371/journal.pdig.0000811 (PMC11978028; doi:10.1371/journal.pdig.0000811)
Supplement: Attachment — Submitted filename: Response_Table.pdf [file pdig.0000811.s004.pdf]

Response to Reviewers

Reviewer	Comment	Our Response	Modification in manuscript	Location
1	The manuscript could benefit from additional detail regarding data preprocessing (i.e., handling of demographics imbalances, if present) and description of software and tools, or programming languages used in data analysis.	**Agree**	Thank you for the feedback. In response to your suggestion, we have strengthened the part pertaining to data preprocessing. We have also mentioned the specific tools we used in the methods section, which is critical for reproducibility.<br><br>**Before**: *"To minimize confounding factors in selecting the patches, the negative patches were carefully sampled to match the positive patches in terms of size, location within the image, patient demographics, and imaging features."*<br><br>**After**: *"We employed a stratified down-sampling approach to minimize confounding factors in patch selection. Given that the pool of patients available for negative patch extraction is much larger than that for positive patches, we carefully subsampled negative patches to ensure that the patient distribution used for their extraction closely matched that of positive patches. Specifically, this process was to control for key confounders, including patient demographics (e.g., race), imaging features, and breast density, preventing the model from exploiting unintended differences in patient composition rather than learning clinically relevant imaging patterns."*<br><br>**Newly Added**: *"All data preprocessing and deep learning model development were conducted in a Python environment using TensorFlow. Statistical analyses were performed using the statsmodels package in Python and cross-validated using StataIC 15.1 (StataCorp, College Station, TX, USA)."*	Methods<br>-Second Paragraph

1	Including references that discuss practical implications for end-users (e.g., radiologists) on how these model errors impact real-world diagnostic decisions or patient outcomes could strengthen the study's relevance.	**Agree**	Thank you for your valuable suggestion. We have added the following sentence to the discussion section:<br><br>*"Recent studies indicate that even modest misclassifications by deep learning models can disrupt radiologists' diagnostic workflows and influence patient management decisions (45, 46). This work was part of ongoing efforts to enhance the clinical utility and fairness of deep learning-based mammography screening by identifying these error patterns in a rigorous way."*	Discussion<br>-First Paragraph
1	Consider reviewing the following reformulated sentences.	**Agree**	We deeply appreciate the effort you put into providing these well-reformulated sentences. We have carefully reviewed your suggestions and made sure to incorporate them into the revised version of our manuscript, which significantly improved the quality of the manuscript.<br><br>**Before**: Breast cancer is the most common cancer in women and causes 42,000 deaths each year in the United States<br>**After**: Breast cancer is the most common cancer among women, leading to approximately 42,000 deaths annually in the United States.<br><br>**Before**: Many Deep Learning (DL) models have been suggested to facilitate the differentiation between normal and suspected abnormal regions of interest (ROIs)<br>**After**: Deep learning (DL) models are increasingly utilized to enhance the differentiation between normal and suspected abnormal regions of interest (ROIs) in mammograms.<br><br>**Before**: The dataset includes detailed demographics, imaging features, pathologic outcomes, and Breast Imaging Reporting and Data System (BI-RADS) tissue densities and scores for both screening and diagnostic exams.	Introduction<br>-First Paragraph<br><br>Introduction<br>-Second Paragraph<br><br>Method<br>-First Paragraph

			**After**: The dataset provides comprehensive detailed demographics, imaging features, pathologic outcomes, and Breast Imaging Reporting and Data System (BI-RADS) tissue densities and scores for both screening and diagnostic exams.<br><br>**Before**: … and enable the identification of populations that may face disadvantages due to the implementation of such tools if no measures are taken.<br>**After**: … and contribute to fairer and more interpretable decision-making models in mammography while identifying populations that may face disadvantages if no measures are taken.	Conclusion<br>-First Paragraph
1	In the caption of Figure 2, add a brief description of the matching process (mentioning the row- and column-wise distribution)	**Agree**	Thank you for the feedback. We have elaborated the process in the caption as follows:<br><br>**Newly Added**: *"The matching process utilized row-wise and column-wise distribution along with overall pixel distribution to maximize similarity in the overall intensity distributions between negative and positive patches."*	Figure 2
1	In line 9 of the Discussion paragraph, provide also literature examples that support your sentence (i.e., classes are interrelated)	**Agree**	Thank you for the suggestion. The relevant literature supporting the interrelation of classes was introduced in the Introduction section, and I reiterated them in the Discussion to reinforce their relevance in the context of our findings.<br><br>**Updated**: *Most subgroup evaluation considers classes like race, age, imaging features, or pathologic outcomes as independent variables (42-44), when in reality, they are interrelated (28-30).*	Discussion<br>-First Paragraph

1	Consider specifying that White class is significantly more likely for false negative with respect to other races, but not Black	**Agree**	Thank you for the great suggestion. We have added the following sentence to the Results section:<br><br>Newly Added: *"Patients who faced false negatives were significantly more likely to be White compared to other racial groups, but this association was not significant when specifically compared to Black patients."*	Results<br>-Last Paragraph
2	Citation formatting appears inconsistent in a few places use uniform formatting.	**Agree**	Thank you for pointing it out. We have carefully reviewed the citations and corrected minor formatting inconsistencies to ensure uniformity. Please let us know if any specific issues remain.	Reference
2	Figures are critical to understanding such a technical study and should be improved with high resolution.	**Clarification**	Thank you for your feedback. The images were created in high resolution, but it seems that the submission system downscaled them upon upload (35.3 MB => 1.9 MB).<br>We will make sure the high-resolution versions are used for publication.	
2	Confidence intervals for false positive/negative risk rates are not provided. Providing this, if possible, will increase the clarity and reliability of the results.	**Agree**	Thank you for your valuable suggestion. We have updated our Table 4 and 5 by adding 95% CI for OR and RR.	Table 4<br>Table 5
